# Development of medullary thyroid carcinoma on a toxic nodule: about an exceptional case

**DOI:** 10.1093/omcr/omaf178

**Published:** 2025-09-28

**Authors:** Ali Halouache, Lahoussaine Abainou, Hajar Agouri, Mohammed Tbouda, Ismael Nakkabi

**Affiliations:** Endocrinology, Diabetology and Metabolic Diseases Department military hospital Oued Eddahab, Agadir, Morocco; Endocrinology, Diabetology and Metabolic Diseases Department military hospital Oued Eddahab, Agadir, Morocco; Anathomopathology Department military hospital Oued Eddahab, Agadir, Morocco; Anathomopathology Department military hospital Oued Eddahab, Agadir, Morocco; Otolaryngology surgery Department military hospital Oued Eddahab , Agadir, Morocco

**Keywords:** toxic adenoma, thyroid, medullary, carcinoma, calcitonin

## Abstract

We report the case of a 60-year-old hypertensive female patient who presented with an anterior neck swelling associated with local compression signs. TSH levels were in favor of hyperthyroidism. Cervical ultrasound showed two nodules classified as EU-TIRADS III, one of which, located at the lower right pole, was hyperfunctioning on I-123 scintigraphy. After total thyroidectomy, the histopathological study confirmed that the toxic nodule corresponded to a medullary thyroid carcinoma.

Calcitonin levels were 115.6 ng/L (N < 10 ng/L). The staging workup, as well as the MEN screening, were unremarkable. An extensive lymph node dissection was performed, revealing no lymph node involvement.

## Introduction

Medullary thyroid carcinoma (MTC) accounts for 5 to 10% of thyroid cancers. It develops from the parafollicular C cells and has a tumor marker: calcitonin. The most common way it is discovered is through the detection of a thyroid adenoma, which is non-secreting in almost all cases. However, the development of MTC from a toxic nodule is an exceptional situation. We report a case of a toxic nodule, surgically treated, whose anatomopathological study was in favor of MTC.

## Case report

Mrs. T.F., a 60-year-old woman with hypertension treated with dual therapy, consulted for an anterior neck swelling discovered on self-palpation, associated with intermittent dysphonia and dysphagia to solids. She had lost 10 kilograms of weight over the past 3 months and experienced palpitations that worsened with stress.

Clinical examination did not reveal signs of thyrotoxicosis other than hypertension and tachycardia. Cervical examination showed a WHO grade I goiter of elastic consistency; the site of a lower right nodule, painless, mobile on swallowing, and with regular borders. Examination of the lymph node areas did not reveal any lymphadenopathy.

Biological tests showed a suppressed TSH at 0.01 IU/ml (N: [0.35–4.9]) and a normal free thyroxine 4 level at 0.97 ng/dl. There were no signs of autoimmunity.

Cervical ultrasound revealed two nodules: the first and largest was in the lower right pole, oval, isoechoic, heterogeneous, well-defined, measuring 26 × 19 mm, classified as EU-TIRADS 3. The second was in the lower left pole, tissue-like, isoechoic containing cystic loculations, measuring 14 × 10 mm, classified as EU-TIRADS 3.

**Figure 1:**
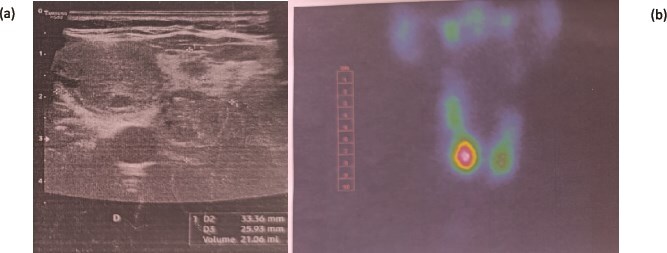
Ultrasound (a) and Tc-99 m thyroid scintigraphy (b) of our patient.

Given these findings, a Tc-99 m thyroid scintigraphy was requested, which showed an appearance consistent with a multinodular goiter with a hot nodule in the lower right lobe, almost completely suppressing the rest of the thyroid parenchyma. (Fig. 1).

**Figure 2:**
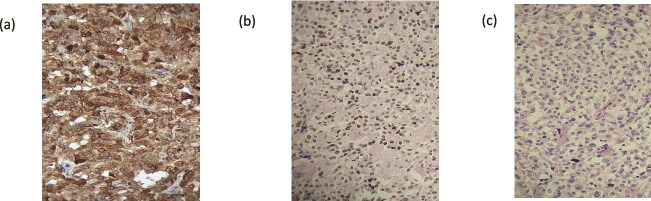
Results of the anatomopathological study of the surgical specimen: (a): Calcitonin antibody ×20, (b): TTF1 antibody ×20, (c): Ki 67 antibody ×20.

Therefore, a total thyroidectomy was decided upon, and the anatomopathological examination of the surgical specimen revealed morphological features and an immunohistochemical profile consistent with a low-grade medullary thyroid carcinoma corresponding to the right lobe nodule (fig. 2).

Calcitonin levels were 115.6 ng/l (N < 10 ng/l), chromogranin A was 182 ng/ml (N < 108), and ACE was 84 ng/ml (N < 4.1).

In the context of a multiple endocrine neoplasia type 2; urinary metoxylated derivatives measured after diagnosis were negative, and the calcium-phosphate balance was normal.

The staging workup (cervico-thoraco-abdomino-pelvic CT scan, bone scan) did not reveal any distant metastases.

A bilateral lymph node dissection was performed, the anatomopathological study of which did not reveal any lymph node metastases (Right neck dissection of groups IIa, III and IV: 0 positive nodes/12 nodes examined; Left neck dissection of groups IIb, IIa, III and IV: 0 positive nodes/11 nodes examined).

Genetic testing for RET gene mutation (sequencing of exons 8, 10, 11, 13, 14, 15, 16 out of 21 exons) was negative.

## Discussion

Toxic thyroid nodules account for 5 to 10% of thyroid nodules [1], defined by increased radiotracer uptake compared to the rest of the thyroid parenchyma. They can be single or multiple (toxic multinodular goiter). The clinical presentation is dominated by the thyrotoxicosis syndrome, but it may be absent. Often, the nodule is discovered incidentally on self-palpation or during cervical imaging. A suppressed TSH leads the diagnosis, which is confirmed by thyroid scintigraphy [2, 3]. According to the recommendations of major learned societies, these nodules are, with very rare exceptions, benign and should not be subjected to fine-needle aspiration, regardless of their size. The diagnosis is based on scintigraphy data, and treatment may be limited to radioiodine therapy [4].

However, the risk of malignancy observed in hot nodules is higher than expected [5]. The most frequent histological type in patients with a hot nodule is papillary carcinoma, followed by follicular carcinoma [6]. Medullary thyroid carcinoma can coexist with hyperthyroidism in 1.6% of cases [7], but its development on a toxic nodule is exceptional, which is a particularity in our patient.

Often, lymphadenopathy is associated with the thyroid nodule in patients with MTC [8]; this clinical sign was not found in Mrs. Fatima’s case.

Basal calcitonin measurement plays an important role in the diagnosis, prognosis, and postoperative follow-up of MTC. Nevertheless, there is a dilemma as to whether or not it should be measured in all patients with a thyroid nodule [9]. In a meta-analysis by Verbeek HHG and colleagues, among 10 000 patients with nodular thyroid disease, 303 had elevated calcitonin levels, and only 23 had MTC. The authors concluded that calcitonin is a sensitive and specific marker for MTC, but its systematic measurement can lead to false positives and unnecessary surgeries; consequently, this measurement should be reserved for cases where the clinical presentation raises suspicion or when thyroid fine-needle aspiration is inconclusive [10]. In contrast, Elisa Giannetta and her team argue that the new immunochemiluminometric assay has improved the reliability and sensitivity of calcitonin and that its measurement should be systematic in all nodular thyroid diseases because it allows for early diagnosis of a potentially more aggressive tumor and improves the sensitivity of cytology (which is less than 50% for MTC) [11]. Although there was a low probability that the nodule seen on ultrasound in our patient was an MTC, especially given its toxic nature, a simple calcitonin measurement would have guided the surgical procedure and avoided a reoperation with the associated risks. The European Thyroid Association (ETA) recommends systematic calcitonin measurement in patients with thyroid nodules [4]. However, this approach has not been adopted by other thyroid associations (ATA, AACE, ACE).

Before proceeding with a total thyroidectomy, the gold standard treatment for MTC, it is important to complete the diagnosis with a staging workup, as well as the search for other components of multiple endocrine neoplasia (MEN), particularly pheochromocytoma, which can be life-threatening if not treated beforehand [12]. In our patient, this workup was not performed preoperatively because the probability of MTC was very low. After anatomopathological diagnosis, this workup did not reveal other components of MEN or other tumor locations.

Total thyroidectomy with central lymph node dissection is considered the standard treatment for MTC [12]. For the same reason mentioned above, this dissection was not performed in our patient, which led to a surgical reintervention, exposing the patient to significant morbidity and mortality [8].

In fact, there is a positive correlation between calcitonin levels and the tumor stage of MTC. In our case, this level exceeded 100 ng/ml, which was suggestive of lateral lymph node involvement [13], thus justifying an extensive lymph node dissection.

A follow-up consultation is scheduled after 3 months. If the calcitonin level becomes undetectable, the surveillance frequency is biannual for the first year, then annually. Otherwise, the calcitonin level will dictate the management. The presence of metastases is probable when it exceeds 150 pg/ml; a level below 10 pg/ml should be supplemented by a stimulation test, and in intermediate cases, this level should be checked after 3 months [14].

## Conclusion

This exceptional clinical case challenges and excludes the prevalent idea that a toxic nodule is invariably benign, thereby emphasizing the importance of systematic calcitonin measurement in the evaluation of any thyroid nodule, including toxic ones, particularly when thyroidectomy is indicated. This would allow us to avoid the need for reoperation and to perform precise preoperative staging. However, this suggestion faces technical barriers related to the cost and availability of this measurement, particularly in our context.
